# Study on the Extraction Method of Microplastic System in Textile Wastewater

**DOI:** 10.3390/polym15061394

**Published:** 2023-03-10

**Authors:** Jiachen Li, Yuanyuan Liu, Yingxi Gao, Xin Li, Yan Gong

**Affiliations:** School of Materials Design and Engineering, Beijing Institute of Fashion Technology, Beijing 100029, China

**Keywords:** microplastics, printing and dyeing wastewater, digestion, density separation, detection and analysis

## Abstract

Microplastic pollution has become a global environmental problem. Textile microplastics are an important component of microplastic pollution, but little is known about their contamination in the industrial environment. The lack of standardized methods for detecting and quantifying textile microplastics is a major obstacle to determining the risks they pose to the natural environment. This study systematically examines the pretreatment options for the extraction of microplastics from printing and dyeing wastewater. The effectiveness of potassium hydroxide, nitric acid–hydrogen peroxide mixed solution, hydrogen peroxide, and Fenton’s reagent for the removal of organic matter from textile wastewater is compared. Three textile microplastics, polyethylene terephthalate, polyamide, and polyurethane, are studied. The effects of the digestion treatment on the physicochemical properties of textile microplastics are characterized. The separation efficiency of sodium chloride, zinc chloride, sodium bromide, sodium iodide, and sodium chloride-sodium iodide mixed solution on the textile microplastics is tested. The results showed that Fenton’s reagent achieved a 78% removal rate of organic matter from printing and dyeing wastewater. Meanwhile, it has less of an effect on the physicochemical properties of textile microplastics after digestion and is the best reagent for digestion. The zinc chloride solution achieved a 90% recovery for separating textile microplastics with good reproducibility. It does not affect the subsequent characterization analysis after separation and is the best solution for density separation.

## 1. Introduction

Currently, researchers have found varying degrees of microplastic pollution in various environments around the world, including the ocean, soil, and atmosphere [1,2,3,4,5]. However, until recently, only 6.3% of studies investigated microplastic pollution in the industrial environment [6]. Textile microplastics are an important component of microplastics, which are predominantly fibrous in form and originate mainly from textiles’ production, processing, use, and disposal processes [7,8,9,10,11]. According to a report published by the International Union for Conservation of Nature (IUCN) in 2017 [12], the total amount of microplastics released into the environment globally is about 1.8–5.0 million tons, of which textile microplastics account for 34.8%. Zhang and Deng et al. [13,14] found high concentrations of textile microplastics in environmental samples near textile enterprises, suggesting that the textile industry is an important potential source of microplastic contamination. Measuring and quantifying textile microplastics on spatial and temporal scales is necessary to characterize their contamination and develop prevention and control measures. However, to date, there are no uniform standard techniques or program guidelines for detecting textile microplastics. Research on textile microplastic detection is still in its infancy, and there is a lack of basic technical references and data sources.

In general, printing and dyeing wastewater is the main medium for the occurrence of textile microplastics [15]. Textile wastewater mainly consists of wastewater generated during textile pretreatment, printing and dyeing, and finishing processes [16]. Depending on the type of fabric to be processed and the dyeing and finishing process, the volume and quality of printing and dyeing wastewater vary. The complexity of printing and dyeing wastewater leads to difficulties in the detection of textile microplastics. Printing and dyeing wastewater usually contains dyes, pastes, auxiliaries, oil agents, acids and bases, fiber impurities, sand substances, inorganic salts, and other substances [17,18]. Currently, the pretreatment process for microplastic detection is generally divided into two parts: removal of organic impurities and separation of microplastics [19,20]. Digestion is often used for the removal of organic matter from samples. Common digestion methods include acid digestion (nitric acid (HNO_3_), perchloric acid (HClO_4_)), alkali digestion (potassium hydroxide (KOH), sodium hydroxide (NaOH)), oxidative digestion (hydrogen peroxide (H_2_O_2_), Fenton’s reagent), and enzymatic digestion [21,22,23,24]. Among them, enzymatic digestion is generally used for less complex environmental samples [25]. In contrast, the organic mixture in printing and dyeing wastewater is more complex and diverse. The removal of organic matter may require multiple types of enzymes to achieve, leading to higher experimental costs and complex steps. The chemical digestion method has a broader specificity and may have better prospects for application to dyeing and printing wastewater. The density separation method is a physical method for separating microplastics from environmental samples [26]. The method is performed by thoroughly mixing a high-density salt solution with the sample and separating it with the help of the density difference between the microplastics and the solution. The density separation method is simple to experiment with and easy to operate. At present, the solutions that have been used for the separation of microplastics are sodium chloride (NaCl, 1.2 g·cm^−3^), zinc bromide (ZnBr_2_, 1.71 g·cm^−3^), zinc chloride (ZnCl_2_, 1.7 g·cm^−3^), and sodium iodide (NaI, 1.8 g·cm^−3^) [27,28,29,30]. Although the application of the density separation method in the detection of microplastics is relatively mature, its effectiveness for the separation of textile microplastics has yet to be verified. It is worth mentioning that most of the studies have chosen to use the digestion and density separation methods to pretreat the samples, but few studies have investigated the effectiveness of the application of these methods. There are differences in the chemical structures and physical properties of different types of microplastics, and improper handling of the pretreatment process can also impact the study’s final results [31,32]. For example, some microplastics are degraded or fragmented under certain digestion conditions, and some denser microplastics cannot be effectively separated in low-density solutions [25,33,34].

This study aims to systematically explore and validate the extraction of microplastics from textile wastewater. The effect of different digestion methods on the removal of organic matter from textile wastewater is tested. Additionally, three textile microplastics, polyester (polyethylene terephthalate, PET), nylon (polyamide, PA), and spandex (polyurethane, PU), are studied to investigate the effects of different digestion protocols and density separation solutions on the detection of textile microplastics. The suitability of the pretreatment methods is verified by analyzing the data on the recovery, morphological, and structural changes of the microplastics before and after the treatment. The results of the study can provide a reference for the detection of microplastics in textile wastewater.

## 2. Materials and Methods

The study on the extraction methods of microplastics from textile wastewater contains two parts. (1) The effect of different digestion methods on the removal of organic matter from printing and dyeing wastewater and on the physicochemical properties of textile microplastics; (2) the separation efficiency of different density separation solutions on textile microplastics.

### 2.1. Materials and Reagents

#### 2.1.1. Microplastic Samples

China is the world’s leading textile producer and processor, and its chemical fiber processing accounts for 70% of the total global production [35]. Among them, polyester (PET), nylon (PA), and spandex (PU) account for 90%, 7%, and 1.5% of the total processed chemical fibers, respectively [36]. Therefore, the study is carried out with three types of microplastics, PET, PA, and PU, as indicative samples. The microplastic samples were purchased from Shanghai Guanbu Mechanical and Electrical Technology Co. The microplastics were all irregularly particulate in shape, colorless, and transparent, with particle sizes ranging from 50 to 120 μm. The study was conducted using Fourier transform infrared spectroscopy (FTIR) to verify the types of polymers and the characteristic infrared absorption peaks.

#### 2.1.2. Textile Wastewater Samples

**Figure 1:**
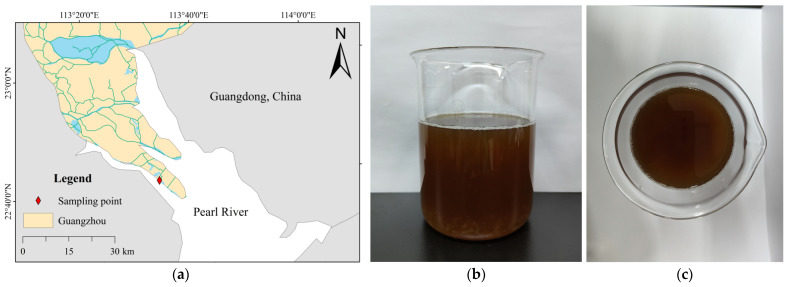
(**a**) Printing and dyeing water sample sampling location; (**b**,**c**) Printing and dyeing wastewater water samples.

The textile wastewater used for the test came from a large textile company (Hutai Textile Co., Ltd., Guangzhou, China) in Guangdong Province, China (Figure 1). The company has a full textile production line for knitting, dyeing, printing, and finishing. The total annual production and processing of textiles in the enterprise are 87,000 tons, and the daily treatment capacity of the wastewater treatment plant on the enterprise’s campus is 20,000 tons [37]. The study was carried out with on-site sampling and encapsulation at the inlet of the wastewater treatment plant, followed by experiments and tests in the laboratory. The sampling time was from 20 July 2021 to 30 July 2021.

The textile wastewater water sample is a dark brown turbid liquid. The chemical oxygen demand (COD) of the wastewater is 700–900 mg·L^−1^ and the pH was 6–10 [37].

### 2.2. Digestion Treatment

#### 2.2.1. Removal of Organic Matter in Printing and Dyeing Wastewater

Chemical oxygen demand (COD) is an important indicator to characterize the organic pollution of water bodies, which can reflect the degree of organic pollution in water bodies. COD is the amount of oxidant consumed when a strong oxidant is used to treat water samples under certain conditions [38]. Although the reducing substances in water are various organic substances, nitrites, sulfides, ferrous salts, etc., the main part of them is organic substances [14]. Therefore, the study was conducted to calculate the digestion efficiency from the COD data before and after the digestion of printing and dyeing wastewater. The initial selection of digestion reagents was based mainly on literature data documenting the effects of digestion efficiency and microplastics [39,40,41]. The digestion reagents tested in the study included potassium hydroxide (KOH, 10% *w*/*v*), nitric acid–hydrogen peroxide mixed solution (HNO_3_, 68% *w*/*v*; H_2_O_2_, 30% *w*/*v*), hydrogen peroxide (H_2_O_2_, 30% *w*/*v*), and Fenton’s reagent (hydrogen peroxide + ferrous sulfate: H_2_O_2_, 30% *w*/*v* + FeSO_4_, 0.05 mol·L^−1^) [21,22,23,24]. The above reagent drugs were purchased from Beijing Tongguang Fine Chemical Company.

Potassium hydroxide, nitric acid–hydrogen peroxide mixed solution, and hydrogen peroxide digestion: Take 500 mL of printing and dyeing wastewater, add 50 mL of digestion reagent, and digest under the conditions of set experimental parameters (a. 25 °C, 24 h; b. 50 °C, 24 h; c. 25 °C, 72 h).

Fenton’s reagent digestion: Take 500 mL of printing and dyeing wastewater, add 25 mL of hydrogen peroxide (H_2_O_2_, 30% *w*/*v*) and 25 mL of ferrous sulfate solution (FeSO_4_, 0.05 mol·L^−1^), adjust the pH to 3 with concentrated sulfuric acid (H_2_SO_4_, 95% *v*/*v*), and digest under the conditions of set experimental parameters (a. 25 °C, 24 h; b. 50 °C, 24 h; c. 25 °C, 72 h) with ice bath control temperature.

Both the original water samples and the digested water samples were tested for COD index, and the detection method was based on the HJ/T 399–2007 rapid digestion spectrophotometric method [42], with a sample size of 3 for each group of data.

#### 2.2.2. Digestion of Textile Microplastics

To further understand the effect of the digestion method on the physicochemical properties of textile microplastics, 0.5 g of textile microplastics was added to 500 mL of ultrapure water, and then the textile microplastics were digested by using potassium hydroxide, nitric acid–hydrogen peroxide mixed solution, hydrogen peroxide, and Fenton’s reagent, according to the digestion method in Section 2.2.1, respectively. After the digestion was finished, the microplastics and the digestion solution were passed through a polytetrafluoroethylene filter membrane (pore size 0.45 μm, Haiyan New Oriental Plastic Technology Co., Ltd., Jiaxing, China) together and rinsed with ultrapure water. After that, the membrane was transferred to a glass dish and dried at 90 °C. The masses of the filter membranes and Petri dishes before and after the addition of microplastic particles were weighed separately, and the recovery of microplastics (Re) was calculated according to Equation (1), and the number of samples for each group of data was three.

(1) Re=M2−M1M0×100%

where Re is the recovery of microplastics (%), *M*_2_ is the total mass of microplastic, filter membrane, and Petri dish after digestion and drying (g), *M*_1_ is the mass of filter membrane and Petri dish before digestion (g), and *M*_0_ is the mass of added microplastic (g).

The microplastics were characterized using Fourier transform mid-infrared spectrometer (FT-MIR, wave number range 400–4000 cm^−1^, resolution 8 cm^−1^, scan number 32, Thermo Fisher Scientific, Waltham, USA), XSP-8CA optical microscope (Shanghai Optical Instrument Factory, Shanghai, China), and 8700LDIR (wave number range 975–1800 cm^−1^, resolution 8 cm^−1^, scan number 64, Agilent Technologies Inc., California, USA) before and after digestion to determine the effect of the digestion method on the infrared characteristic absorption, morphology, and particle size of the microplastics.

### 2.3. Density Separation Processing

Density separation is a simple, economical, and efficient method for separating microplastics [28]. The density of textile wastewater was tested to be 1.11 g·cm^−3^. To facilitate the control of variables, the study tested the separation efficiency of different density separation media on textile microplastics using ultrapure water as a substrate. A total of five density separation media were tested in the study: sodium chloride solution (NaCl, 1.17 g·cm^−3^), zinc chloride solution (ZnCl_2_, 1.68 g·cm^−3^), zinc bromide solution (ZnBr_2_, 1.71 g·cm^−3^), sodium iodide solution (NaI, 1.78 g·cm^−3^), and sodium chloride-sodium iodide mixture (1:1 *v*/*v*, 1.56 g·cm^−3^) [22,27,28,29]. All the above reagents were analytically pure and purchased from Beijing Tongguang Fine Chemical Company. To ensure the accuracy of the density separation solution, the salt solutions used for the tests were subjected to density checks.

Density separation experiment: Add 0.5 g of textile microplastics to 500 mL of density separation solution, mix well, and transfer the solution to a separatory funnel for 24 h. When the resting period is over, pass the upper layer of the solution in the partition funnel through a polytetrafluoroethylene filter membrane (pore size 0.45 μm, Haiyan New Oriental Plastic Technology Co., Ltd., Jiaxing, China) and rinse with ultrapure water. The filter membrane was transferred to a glass dish and dried at 90 °C. The masses of the filter membranes and Petri dishes before and after the addition of microplastic particles were weighed separately, and the recovery of microplastics (Re) was calculated according to Equation (1), with a sample size of three for each group of data.

### 2.4. Quality Control

In order to avoid contamination of samples during the experimental process, the following contamination control measures were taken during the experimental operation: laboratory personnel wore clean cotton lab coats and cotton masks; all operating surfaces, equipment, and glass equipment needed to be cleaned with ethanol and deionized water before use.

### 2.5. Data Processing

The experimental data were processed, and graphs were drawn using Excel 2020, SPSS 19.0, Origin 2021, and other software.

## 3. Results and Discussion

### 3.1. Removal of Organic Matter from Printing and Dyeing Wastewater

**Figure 2:**
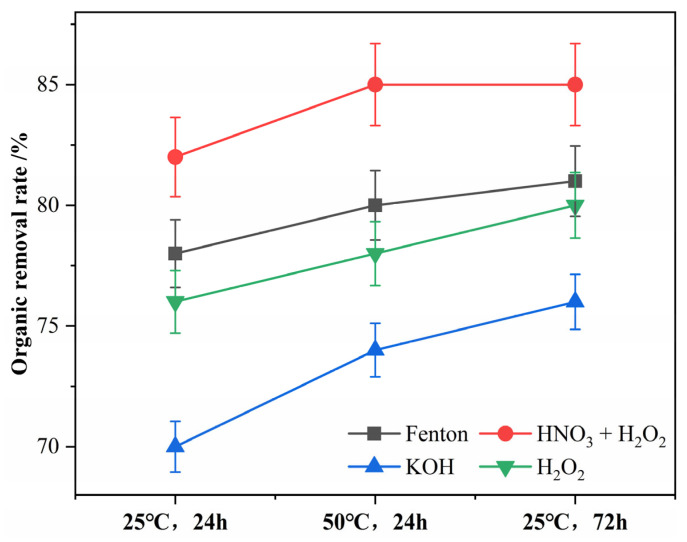
Removal rate of organic matter from printing and dyeing wastewater by digestion methods.

The removal rates of organic matter from textile wastewater by different digestion methods are shown in Figure 2. Under the experimental conditions of “25 °C, 24 h”, the highest organic removal rate of 82.4% was achieved for the nitric acid–hydrogen peroxide mixture. This is mainly due to its higher acidity and oxidizing properties [25,40]. In contrast, the reagent with the lowest organic removal rate was potassium hydroxide, with only 70.2% organic removal. Potassium hydroxide and Fenton’s reagent had a relatively similar effect on the removal of organic matter from printing and dyeing wastewater, and the experimental data were relatively close (76–78%). A study by Akyildiz et al. [19] also showed that Fenton’s reagent, hydrochloric acid, and hydrogen peroxide were more effective than potassium hydroxide for removing organic matter from textile wastewater, which is in agreement with the results of the present study. With the increase in digestion temperature and digestion time, the organic removal rates of different digestion reagents were improved. Among them, the enhancement of the time factor was slightly higher, and the organic removal rate of each reagent increased by 4.0% on average, while the enhancement rate of the temperature factor also reached 2.7%. As suggested by Pfeiffer and Munno et al. [41,43], increasing the digestion temperature is an effective way to facilitate the removal of organic matter from the sample. However, exposure of some polymers to the digestion solution under prolonged or high-temperature conditions may lead to their dissolution. Therefore, the study recommends that the temperature of digestion should not exceed 60 °C and the duration of digestion should not exceed 72 h when performing digestion operations on textile printing and dyeing wastewater.

The nitric acid–hydrogen peroxide mixed solution, hydrogen peroxide, and Fenton’s reagent selected for the study were able to remove most of the organic matter (>75%) from the printing and dyeing wastewater, which is in agreement with the results of previous studies [19,44]. Additionally, a study by Freya et al. [45,46,47] showed that oxidizing digestion agents are more effective in removing organic matter from textile wastewater. Therefore, the study concluded that both Fenton’s reagent and hydrogen peroxide are suitable digestion methods for removing organic matter from textile wastewater. However, additional attention needs to be paid to the exothermic reaction when using Fenton’s reagent, and the temperature needs to be monitored and controlled.

### 3.2. Effect of Digestion Method on the Physicochemical Properties of Microplastics

#### 3.2.1. Recovery of Textile Microplastics

**Figure 3:**
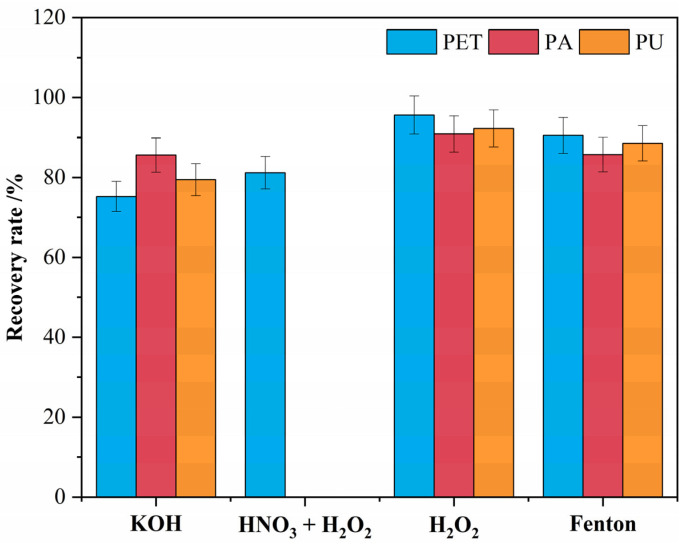
Recovery of textile microplastics after digestion treatments.

The recovery of PET, PA, and PU microplastics by different digestion methods is shown in Figure 3.

In the potassium hydroxide solution, the recovery of PA was 85.6%, while the recovery of PET and PU was only 75.2% and 79.4%. It was concluded that the main reason for the low recovery of PET and PU microplastics was the hydrolysis of ester bonds in the molecular structure of PET and PU, while potassium hydroxide had less of an effect on the amide bonds in the molecular structure of PA, so its recovery was at a high level [48]. In the nitric acid–hydrogen peroxide mixed solution, PA and PU will be dissolved, and there is almost no recovery. Since PET has some acid resistance, partial recovery of PET exists. Compared to acid and alkali digestion, oxidative digestion has less effect on the stability of textile microplastics [40,49]. In the hydrogen peroxide solution, there was essentially no mass loss of PET, PA, and PU, and the recoveries were all at a high level, with an average recovery of 92.3%. Similarly, in Fenton’s reagent solution, the average recovery of the three textile microplastics reached 88.7%, which is within the acceptable range. Treilles et al. [50] tested the effect of different digestion agents on textile fiber microplastics. They found that potassium hydroxide digestion agents have a greater degradation effect on polyester-like fibers, while oxidative digestion is better at maintaining fiber integrity. Therefore, hydrogen peroxide solution and Fenton’s reagent are more suitable digestion methods, considering digestion recovery. If acid and alkali digestion solutions are to be used, the experimentalists need to pay extra attention to the loss of samples.

**Figure 4:**
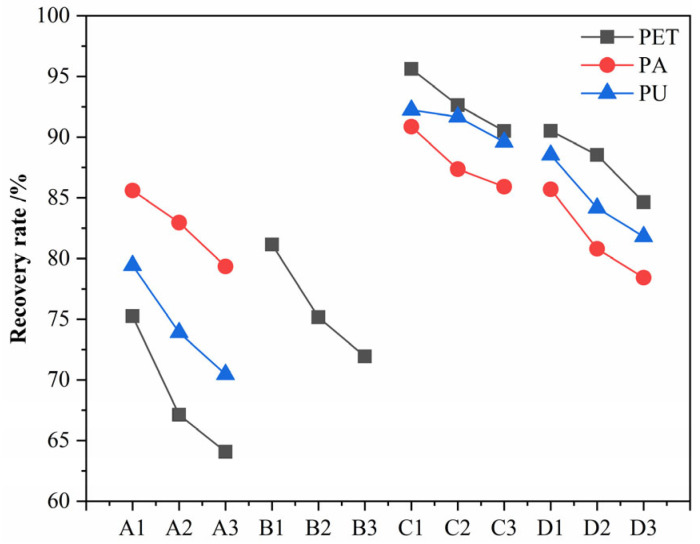
Recovery of textile microplastics at different digestion temperatures and times (A~D denote: four digestion methods of KOH, HON_3_ + H_2_O_2_, H_2_O_2_, and Fenton, respectively; 1~3 denote: three digestion parameters of 24 h digestion at 25 °C, 24 h digestion at 50 °C, and 72 h digestion at 25 °C, respectively).

Increasing the digestion temperature and extending the digestion time are common means to improve digestion efficiency [51,52]. However, for microplastics, changes in temperature and time can also have a large impact on their stability. As shown in Figure 4, the recovery of all three textile microplastics showed a further decrease after increasing the digestion temperature or extending the digestion time, which is in line with the findings in the literature [43]. Pfeiffer et al. [41] found that increasing the digestion temperature from 20 to 60 °C significantly improved the digestion of acid, base, and oxidation digestion reagents. In the potassium hydroxide solution, the recoveries of PET and PU decreased by 10.8% and 6.9%, respectively, with the increase in the digestion temperature. It was hypothesized that the temperature increase led to a further increase in the hydrolysis of ester bonds in the molecular structure of both microplastics, resulting in mass loss, as also mentioned by Radford et al. [45] in his study. PET was also more affected in the nitric acid–hydrogen peroxide mixture, with a 7.4% decrease in the recovery of microplastics. Only in the hydrogen peroxide and Fenton’s reagent solutions did the increase in temperature have a smaller effect on the stability of the textile microplastics, with a smaller decrease of about 3%. It is believed that this may be due to the exothermic reaction of the oxidizing digestant during the digestion process, which would result in a lesser effect of the artificially increased reaction temperature on the digestion effect [43]. The increase in digestion temperature does improve digestion efficiency. However, increasing the temperature also amplifies the digestion solution’s effect on the microplastics’ stability. The results show that the increase in digestion time has a more severe effect on textile microplastics’ stability than the digestion temperature. At present, there are fewer studies on digestion time’s effect on microplastics’ stability. In potassium hydroxide, nitric acid–hydrogen peroxide, and Fenton’s reagent solutions, increasing the digestion time resulted in 14%, 11%, and 7% decreases in the recovery of microplastics, respectively. The microplastic stability was less affected in the hydrogen peroxide solution, and the recovery rate decreased by only 4%. According to the experimental results in 3.1, the effect of digestion time on the removal of organic matter from wastewater is slightly higher than that of digestion temperature, but the effect on textile microplastics is more serious. Therefore, in the testing of textile microplastic detection, the study does not recommend enhancing digestion efficiency by extending digestion time and choosing the appropriate digestion solution, and the digestion temperature is a more reasonable digestion idea.

#### 3.2.2. Spectral Changes of Textile Microplastics

**Figure 5:**
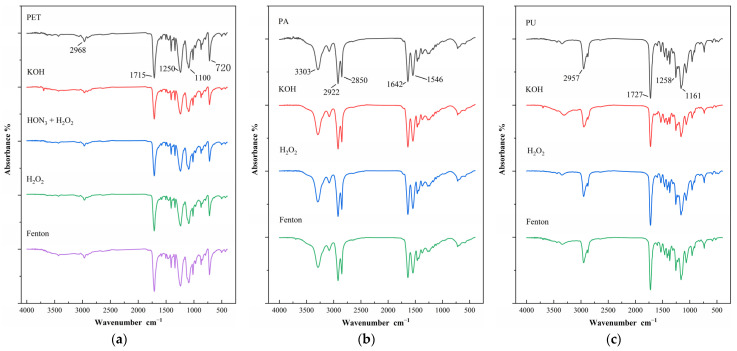
(**a**) Infrared spectra of polyethylene terephthalate (PET) microplastics before and after digestion; (**b**) Infrared spectra of polyamide (PA) microplastics before and after digestion; (**c**) Infrared spectra of polyurethane (PU) microplastics before and after digestion.

The study was carried out to analyze and characterize the textile microplastics after the digestion treatment using Fourier variation infrared spectroscopy to confirm whether the digestion process affected the textile microplastics’ chemical properties. The results showed that the different digestion methods did not affect the chemical properties of the textile microplastics, the infrared characteristic absorption wavelengths of all three textile microplastics did not change significantly before and after digestion (Figure 5), and the experimental results are consistent with the findings in the literature [19,50,53].

Similar to sodium hydroxide, a potassium hydroxide solution would hydrolyze the ester bonds in the molecular structure of PET and PU during the digestion process, causing the signal intensity of IR characteristic absorption to be weakened [40]. For example, the peak intensities of the symmetric stretching vibration peaks of-C-O-C near 1250 cm^−1^ and 1100 cm^−1^ and the stretching vibration peak of-C=O near 1715 cm^−1^ were weakened. However, the wavelengths of the infrared characteristic absorption of PET and PU did not change significantly, which means that some of the lost particles have been degraded or dissolved in the digestion solution, and the properties of the remaining microplastic particles are not affected [41]. However, Herrera et al. [54] did not observe any adverse effects on PET particles when using potassium hydroxide for digestion. It is believed that this may be due to the use of large particle-size microplastics in the above study, which have a low surface area-to-mass ratio, making them less susceptible to the effects of the digestion reagent. This, coupled with the very small number of particles used in the above study, can seriously reduce the reliability of their results [50]. In the nitric acid–hydrogen peroxide mixed solution, PA and PU will be dissolved, and the molecular structure will be destroyed, while the signal intensity of infrared characteristic absorption of PET is slightly weakened, but it does not affect its detection and identification. In the hydrogen peroxide solution and Fenton’s reagent solution, the infrared characteristic absorption wavelengths of the three textile microplastics did not change significantly before and after the dissolution. From the experimental results, the spectra of the digested-treated microplastic particles showed only small changes, compared to the spectra of the non-digested-treated particles. Therefore, except for the case where the microplastics were dissolved, the acid, alkali, and oxidative digestions did not affect the qualitative analysis of the textile microplastics.

#### 3.2.3. Morphological Changes of Textile Microplastics

**Figure 6:**
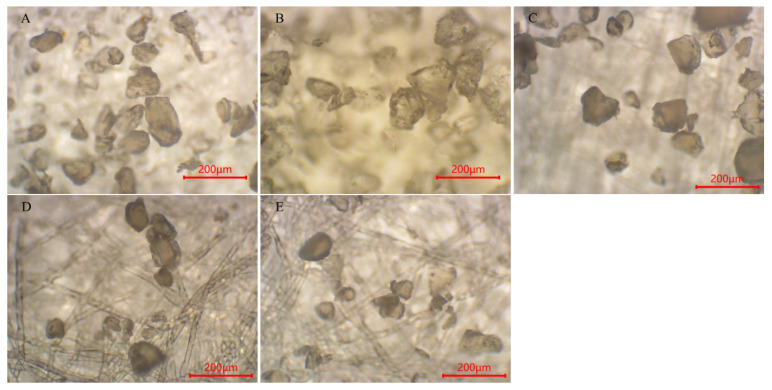
Morphological images of polyethylene terephthalate (PET) before and after digestion (**A**–**E** denote: not ablated, KOH, HON_3_ + H_2_O_2_, H_2_O_2_, Fenton, respectively).

**Figure 7:**
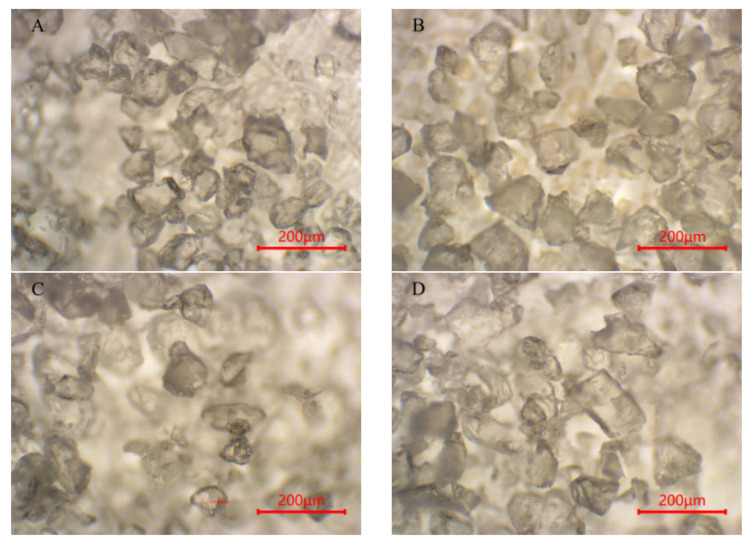
Morphological images of polyamide (PA) microplastics before and after digestion (**A**–**D** denote: not ablated, KOH, H_2_O_2_, Fenton, respectively).

**Figure 8:**
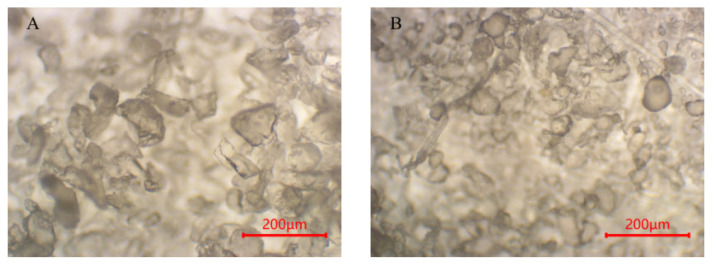

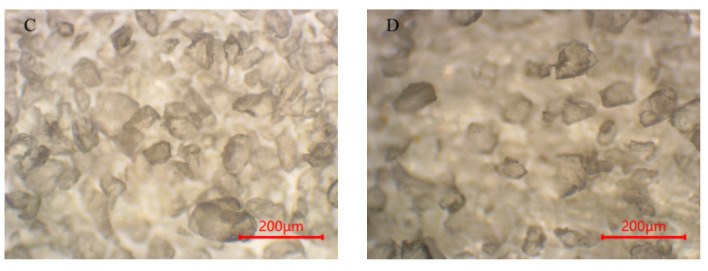
Morphological images of polyurethane (PU) microplastics before and after digestion (**A**–**D** denote: not ablated, KOH, H_2_O_2_, Fenton, respectively).

Although digestion’s effect on the textile microplastics’ chemical properties was small, the effect on their particle size and morphology was more pronounced. To further investigate the effect of digestion on the morphology of textile microplastics, the study used LDIR8700 and optical microscopy to characterize and analyze the microplastics before and after digestion (Figure 6, Figure 7 and Figure 8).

For the PET particles, the particle size of the microplastics increased after the digestion of both the potassium hydroxide solution and the nitric acid–hydrogen peroxide mixture, which, combined with the decrease in the number of small-sized microplastics in the microscopic images, was presumed to be due to the cross-linking or agglomeration of the PET particles as a result of the digestion [55,56]. The PET pellets’ morphology was unchanged after the peroxide solution and Fenton’s reagent digestion treatment and remained irregularly shredded, with the overall particle size unaffected. For PA pellets, dissolution occurred during the digestion of the nitric acid–hydrogen peroxide solution, and there was almost no recovery. After the digestion of potassium hydroxide, hydrogen peroxide, and Fenton’s reagent, the particle size data (Table 1) showed that the PA particles all increased in size, but the morphology was almost unchanged. It is worth mentioning that Treilles et al. [50] found in their study that hydrogen peroxide affects the fiber properties (toughness and elongation at break) of PA. At the same time, potassium hydroxide and Fenton’s reagent did not affect its fiber properties. For the PU particles, Figure 8A shows the PU particles without digestion, and some of the particles have angular edges. After the digestion treatment with potassium hydroxide solution, the angles of some particles disappeared, and the shape was close to spherical, with some particles agglomerated and cross-linked, as shown in Figure 8B. At the same time, from the change in particle size, the particle size of PU particles decreased, presumably because the potassium hydroxide solution led to the hydrolysis of part of the structure of PU particles [40]. Similar to PET particles, the morphology of PU particles changed little after the hydrogen peroxide solution and Fenton reagent digestion treatment, and the overall particle size was not affected. In summary, the acid and alkali digestion methods have a certain influence on the morphology and particle size of textile microplastics, which will make the final microplastic data deviated, while the oxidation digestion method has less influence on the morphology and particle size of textile microplastics, which can ensure the accuracy and reliability of the test data.

### 3.3. Density Separation of Textile Microplastics

**Figure 9:**
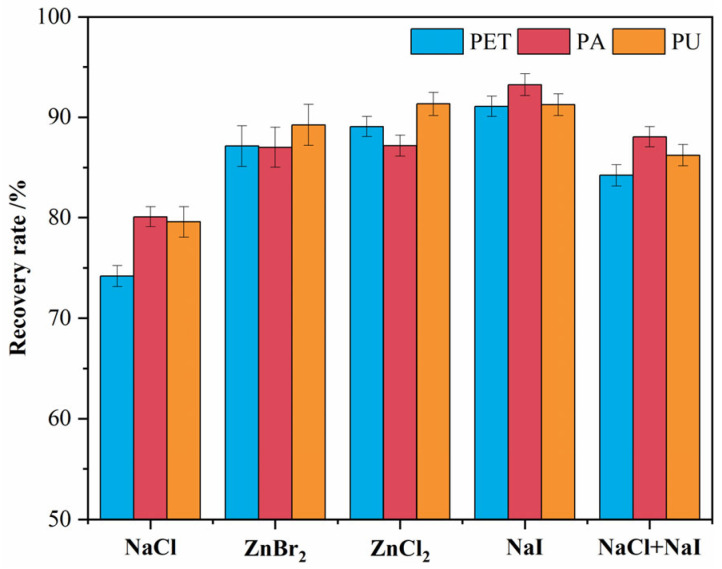
Separation and recovery of textile microplastics by density separation solutions.

The spiked recoveries of different density separation solutions for textile microplastics are shown in Figure 9. Quinn and Coppock et al. [27,29] similarly tested the effectiveness of different density solutions for separating different types of microplastics. Currently, there are abundant methods for the separation and extraction of microplastics, there are differences in the testing methods and experimental samples among studies, and the data results are less comparable between studies. For textile microplastics, the experimental results of this study are as follows.

Among the tested density solutions, the separation recovery of NaCl for all three textile microplastics was at a low level. Since the density of PET microplastics was greater than that of NaCl solution, its separation recovery in NaCl solution was only 74.2%, which is in accordance with the literature [24,26]. However, NaCl solution also has the characteristics of low cost, easy accessibility, and environmental friendliness [57], which can be preferred when performing the separation of low-density polymers.

**Figure 10:**
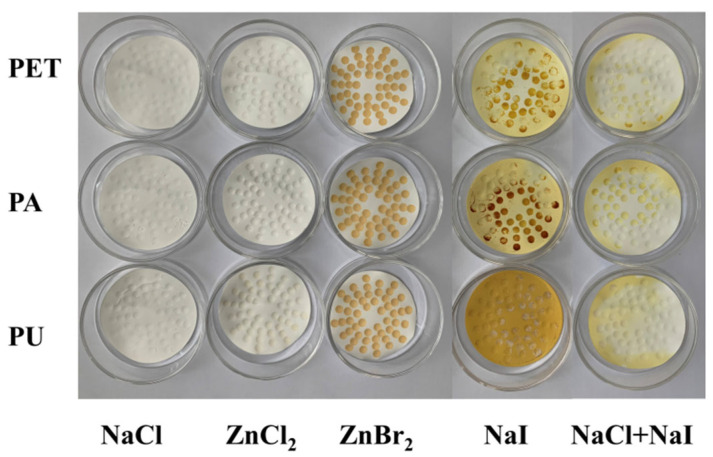
Filter membranes after the use of density separation solutions.

The NaI solution showed the highest recovery of 91.2% (PET), 93.7% (PA), and 91.7% (PU) for the separation of textile microplastics among all tested solutions. However, NaI is relatively expensive, and its solution is hazardous to the environment [58]. In addition, during the testing process, it was also found that NaI is highly oxidizing, and its use can lead to oxidation of the filter membranes to yellow and black colors, which has a greater impact on the subsequent visual identification and qualitative analysis (Figure 10). Nuelle et al. [28] also showed that, although NaI solution can extract all types of microplastics, it is very expensive, and the oxidizing conditions can limit its application. Therefore, the study attempted to mix NaI and NaCl to separate and recover textile microplastics. The results showed that the recovery of the mixed solution was between the two solutions, which indicated that the efficiency could be improved by increasing the solution density. Additionally, the oxidation of the NaI solution on the filter membrane was moderated by the addition of NaCl, and the experimental cost was reduced (Figure 10). At present, there are still more uncertainties in the mixed solutions used for the separation of microplastics, for example, the ratio of solutions, dosage, cost, etc. More systematic research and exploration are expected to follow.

ZnCl_2_ and ZnBr_2_ solutions have been applied in recent years for the separation of microplastics [27,29]. Among them, the separation recoveries of ZnCl_2_ solution for textile microplastics were 89.1% (PET), 87.2% (PA), and 91.3% (PU), which were slightly higher than those of ZnBr_2_ solution. However, the error bars of the experimental data of the ZnBr_2_ solution had a larger range, i.e., the separation reproducibility was poor. Additionally, the ZnBr_2_ solution would leave brown material on the filter membrane after flotation, which would affect the subsequent visual identification and qualitative analysis (Figure 10). In contrast, the separation effect of the ZnCl_2_ solution is more stable and does not interfere with the subsequent assay after flotation. Therefore, the study recommended the use of ZnCl_2_ solution in textile wastewater. However, when ZnCl_2_ is used, the environmental impact of the solution must be carefully considered, and certain precautions need to be taken to reduce its impact [59]. In addition, the study tested simulated wastewater, and the above method needs to be further adapted to consider the environmental samples’ specificity; for example, microplastics may be encapsulated in the environmental samples by other materials, which can further reduce the efficiency.

## 4. Conclusions

This study is the first to systematically compare different methods for the digestion and density separation of textile microplastics from textile wastewater, and it emphasizes the importance of considering sample characteristics when selecting a method for extracting microplastics. In the digestion experiments, potassium hydroxide and hydrogen peroxide had low removal rates of organic matter from dyeing wastewater. At the same time, nitric acid destroyed the dissolved part of textile microplastics, and only Fenton’s reagent could ensure the digestion efficiency without affecting the physicochemical properties of textile microplastics. In the density separation experiments, NaCl could not effectively separate high-density microplastics. NaI and ZnBr_2_ had residues on the filter membrane after separation, while ZnCl_2_ could meet the separation and recovery of high-density microplastics. Meanwhile, the separated filter membrane did not affect the subsequent analytical characterization. Therefore, the study suggests using Fenton’s reagent and ZnCl_2_ for experiments in the study of microplastic detection in textile wastewater. Considering the method efficiency in the detection and analysis of microplastics and using the calibration of relevant studies to ensure the precision of the study will help us to better understand microplastic contamination in textile wastewater.

## Figures and Tables

**Table 1:** Particle size changes of microplastic particles before and after the digestion treatment.

Digestion Processing	Particle Size/μm		
	Polyethylene Terephthalate (PET)	Polyamide (PA)	Polyurethane (PU)
Original sample	82.21 ± 27.04	83.75 ± 24.73	86.74 ± 27.80
Potassium hydroxide	134.88 ± 34.31	108.36 ± 31.72	67.92 ± 16.24
Nitric acid + hydrogen peroxide	97.64 ± 28.32	/	/
Hydrogen peroxide	81.00 ± 25.19	101.30 ± 26.70	79.71 ± 28.49
Fenton’s reagent	85.89 ± 29.88	105.27 ± 37.70	84.45 ± 22.17

“/” indicates that the sample was destroyed by dissolution during the digestion.

## Data Availability

The data presented in this study are available on request from the corresponding author.

## References

[1] Suaria G., Achtypi A., Perold V., Lee J.R., Pierucci A., Bornman T.G., Aliani S., Ryan P.G. (2020). Microfibers in Oceanic Surface Waters: A Global Characterization. Sci. Adv..

[2] Amelia T.S.M., Khalik W.M.A.W.M., Ong M.C., Shao Y.T., Pan H.J., Bhubalan K. (2021). Marine Microplastics as Vectors of Major Ocean Pollutants and Its Hazards to the Marine Ecosystem and Humans. Prog. Earth Planet. Sci..

[3] Barrows A.P.W., Cathey S.E., Petersen C.W. (2018). Marine Environment Microfiber Contamination: Global Patterns and the Diversity of Microparticle Origins. Environ. Pollut..

[4] Dris R., Gasperi J., Saad M., Mirande C., Tassin B. (2016). Synthetic Fibers in Atmospheric Fallout: A Source of Microplastics in the Environment?. Mar. Pollut. Bull..

[5] Yan L., Peng W. (2021). Research of New Pollutant Microplastics in Soil. IOP Conference Series: Earth and Environmental Science.

[6] Jin M., Liu J., Yu J., Zhou Q., Wu W., Fu L., Yin C., Fernandez C., Karimi-Maleh H. (2022). Current Development and Future Challenges in Microplastic Detection Techniques: A Bibliometrics-Based Analysis and Review. Sci. Prog..

[7] Zhou H., Zhou L., Ma K. (2020). Microfiber from Textile Dyeing and Printing Wastewater of a Typical Industrial Park in China: Occurrence, Removal and Release. Sci. Total Environ..

[8] Choi S., Kwon M., Park M.J., Kim J. (2021). Analysis of Microplastics Released from Plain Woven Classified by Yarn Types during Washing and Drying. Polymers.

[9] Acharya S., Rumi S.S., Hu Y., Abidi N. (2021). Microfibers from Synthetic Textiles as a Major Source of Microplastics in the Environment: A Review. Text. Res. J..

[10] Zhang Y.Q., Lykaki M., Markiewicz M., Alrajoula M.T., Kraas C., Stolte S. (2022). Environmental Contamination by Microplastics Originating from Textiles: Emission, Transport, Fate and Toxicity. J. Hazard. Mater..

[11] Salvador Cesa F., Turra A., Baruque-Ramos J. (2017). Synthetic Fibers as Microplastics in the Marine Environment: A Review from Textile Perspective with a Focus on Domestic Washings. Sci. Total Environ..

[12] Hale R.C., Seeley M.E., la Guardia M.J., Mai L., Zeng E.Y. (2020). A Global Perspective on Microplastics. J. Geophys. Res. Oceans.

[13] Zhang C., Zhou H., Cui Y., Wang C., Li Y., Zhang D. (2019). Microplastics in Offshore Sediment in the Yellow Sea and East China Sea, China. Environ. Pollut..

[14] Deng H., Wei R., Luo W., Hu L., Li B., Di Y., Shi H. (2020). Microplastic Pollution in Water and Sediment in a Textile Industrial Area. Environ. Pollut..

[15] Xu C., Gu C., Ni Y., Shen C., Wang H., Wu J., Li F. (2021). Occurrence and Release of Fibrous Microplastic from Dyeing and Printing Wastewater. Fangzhi Xuebao/J. Text. Res..

[16] Liu X., Yuan W., Di M., Li Z., Wang J. (2019). Transfer and Fate of Microplastics during the Conventional Activated Sludge Process in One Wastewater Treatment Plant of China. Chem. Eng. J..

[17] Azanaw A., Birlie B., Teshome B., Jemberie M. (2022). Textile Effluent Treatment Methods and Eco-Friendly Resolution of Textile Wastewater. Case Stud. Chem. Environ. Eng..

[18] Bhattacharjee S. (2017). Removal of Biological Organic Matter and Suspended Solid from Textile Wastewater Using Anaerobic-Aerobic Process: A Review of an Industrial Implementation. J. Sci. Res..

[19] Akyildiz S.H., Sezgin H., Yalcin B., Yalcin-Enis I. (2023). Optimization of the Textile Wastewater Pretreatment Process in Terms of Organics Removal and Microplastic Detection. J. Clean Prod..

[20] Biyik Y., Baycan N. (2021). Comparison of Microplastic Detection Methods in Wastewater Treatment Plants. Environ. Sci. Proc..

[21] Ding N., An D., Yin X., Sun Y. (2020). Detection and Evaluation of Microbeads and Other Microplastics in Wastewater Treatment Plant Samples. Environ. Sci. Pollut. Res..

[22] Karami A., Golieskardi A., Choo C.K., Romano N., Ho Y.B., Salamatinia B. (2017). A High-Performance Protocol for Extraction of Microplastics in Fish. Sci. Total Environ..

[23] Dehaut A., Cassone A.-L., Frère L., Hermabessiere L., Himber C., Rinnert E., Rivière G., Lambert C., Soudant P., Huvet A. (2016). Microplastics in Seafood: Benchmark Protocol for Their Extraction and Characterization. Environ. Pollut..

[24] Tirkey A., Upadhyay L.S.B. (2021). Microplastics: An Overview on Separation, Identification and Characterization of Microplastics. Mar. Pollut. Bulletin..

[25] Prata J.C., da Costa J.P., Duarte A.C., Rocha-Santos T. (2019). Methods for Sampling and Detection of Microplastics in Water and Sediment: A Critical Review. TrAC—Trends Anal. Chem..

[26] Stock F., Kochleus C., Bänsch-Baltruschat B., Brennholt N., Reifferscheid G. (2019). Sampling Techniques and Preparation Methods for Microplastic Analyses in the Aquatic Environment—A Review. TrAC—Trends Anal. Chem..

[27] Quinn B., Murphy F., Ewins C. (2017). Validation of Density Separation for the Rapid Recovery of Microplastics from Sediment. Anal. Methods.

[28] Nuelle M.T., Dekiff J.H., Remy D., Fries E. (2014). A New Analytical Approach for Monitoring Microplastics in Marine Sediments. Environ. Pollut..

[29] Coppock R.L., Cole M., Lindeque P.K., Queirós A.M., Galloway T.S. (2017). A Small-Scale, Portable Method for Extracting Microplastics from Marine Sediments. Environ. Pollut..

[30] Ainali N.M., Kalaronis D., Kontogiannis A., Evgenidou E., Kyzas G.Z., Yang X., Bikiaris D.N., Lambropoulou D.A. (2021). Microplastics in the Environment: Sampling, Pretreatment, Analysis and Occurrence Based on Current and Newly-Exploited Chromatographic Approaches. Sci. Total Environ..

[31] Bakaraki Turan N., Sari Erkan H., Onkal Engin G. (2021). Microplastics in Wastewater Treatment Plants: Occurrence, Fate and Identification. Process Saf. Environ. Prot..

[32] He D., Luo Y., Lu S., Liu M., Song Y., Lei L. (2018). Microplastics in Soils: Analytical Methods, Pollution Characteristics and Ecological Risks. TrAC—Trends Anal. Chem..

[33] Corami F., Rosso B., Morabito E., Rensi V., Gambaro A., Barbante C. (2021). Small Microplastics (<100 Μm), Plasticizers and Additives in Seawater and Sediments: Oleo-Extraction, Purification, Quantification, and Polymer Characterization Using Micro-FTIR. Sci. Total Environ..

[34] Bretas Alvim C., Mendoza-Roca J.A., Bes-Piá A. (2020). Wastewater Treatment Plant as Microplastics Release Source—Quantification and Identification Techniques. J. Environ. Manag..

[35] Wang Q., Yang X. (2021). Analysis on Development Process and General Situation of Modern Chinese Textile Technology. Asian Soc. Sci..

[36] Chen M., Chakraborty S., Xiong J., Scaringella L., Descubes I. (2021). Business Model Renewal and Environment Changes: Insights of Chinese Textile Industry. Strateg. Change.

[37] Hutai (Panyu) Textile & Dyeing Co. https://www.pacific-textiles.com/sc/.

[38] Bidu J.M., Njau K.N., Rwiza M., van der Bruggen B. (2023). Textile Wastewater Treatment in Anaerobic Reactor: Influence of Domestic Wastewater as Co-Substrate in Color and COD Removal. S. Afr. J. Chem. Eng..

[39] Constant M., Billon G., Breton N., Alary C. (2021). Extraction of Microplastics from Sediment Matrices: Experimental Comparative Analysis. J. Hazard Mater..

[40] Hurley R.R., Lusher A.L., Olsen M., Nizzetto L. (2018). Validation of a Method for Extracting Microplastics from Complex, Organic-Rich, Environmental Matrices. Environ. Sci. Technol..

[41] Pfeiffer F., Fischer E.K. (2020). Various Digestion Protocols Within Microplastic Sample Processing—Evaluating the Resistance of Different Synthetic Polymers and the Efficiency of Biogenic Organic Matter Destruction. Front Environ. Sci..

[42] HJ/T 399-2007 (2007). Determination of Chemical Oxygen Demand, Water Quality, Rapid Extinction Spectrophotometric Method.

[43] Munno K., Helm P.A., Jackson D.A., Rochman C., Sims A. (2018). Impacts of Temperature and Selected Chemical Digestion Methods on Microplastic Particles. Environ. Toxicol. Chem..

[44] Crawford C.B., Quinn B. (2016). Microplastic Pollutants.

[45] Radford F., Zapata-Restrepo L.M., Horton A.A., Hudson M.D., Shaw P.J., Williams I.D. (2021). Developing a Systematic Method for Extraction of Microplastics in Soils. Anal. Methods.

[46] Tagg A.S., Harrison J.P., Ju-Nam Y., Sapp M., Bradley E.L., Sinclair C.J., Ojeda J.J. (2017). Fenton’s Reagent for the Rapid and Efficient Isolation of Microplastics from Wastewater. Chem. Commun..

[47] Al-Azzawi M.S.M., Kefer S., Weißer J., Reichel J., Schwaller C., Glas K., Knoop O., Drewes J.E. (2020). Validation of Sample Preparation Methods for Microplastic Analysis in Wastewater Matrices-Reproducibility and Standardization. Water.

[48] Cai H., Du F., Li L., Li B., Li J., Shi H. (2019). A Practical Approach Based on FT-IR Spectroscopy for Identification of Semi-Synthetic and Natural Celluloses in Microplastic Investigation. Sci. Total Environ..

[49] Qiu Q., Tan Z., Wang J., Peng J., Li M., Zhan Z. (2016). Extraction, Enumeration and Identification Methods for Monitoring Microplastics in the Environment. Estuar. Coast. Shelf Sci..

[50] Treilles R., Cayla A., Gaspéri J., Strich B., Ausset P., Tassin B. (2020). Impacts of Organic Matter Digestion Protocols on Synthetic, Artificial and Natural Raw Fibers. Sci. Total Environ..

[51] Möller J.N., Löder M.G.J., Laforsch C. (2020). Finding Microplastics in Soils: A Review of Analytical Methods. Environ. Sci. Technol..

[52] Wang Z., Meng Q., Li W., Yu L., Qin Y., Hao W. (2020). Effect of Different Digestion Methods on Microplastic Quality and Surface Characteristics. Chin. J. Environ. Eng..

[53] Cabernard L., Roscher L., Lorenz C., Gerdts G., Primpke S. (2018). Comparison of Raman and Fourier Transform Infrared Spectroscopy for the Quantification of Microplastics in the Aquatic Environment. Environ. Sci. Technol..

[54] Herrera A., Garrido-Amador P., Martínez I., Samper M.D., López-Martínez J., Gómez M., Packard T.T. (2018). Novel Methodology to Isolate Microplastics from Vegetal-Rich Samples. Mar. Pollut. Bull..

[55] Zhong Yingying Z.H.W.T.B.Q.C.G.C.X. (2022). Comparison of Ablation Methods for the Detection of Microplastics in Mussels. J. Food Saf. Qual. Insp..

[56] Pfohl P., Roth C., Meyer L., Heinemeyer U., Gruendling T., Lang C., Nestle N., Hofmann T., Wohlleben W., Jessl S. (2021). Microplastic Extraction Protocols Can Impact the Polymer Structure. Microplastics Nanoplastics.

[57] Galgani F., Hanke G., Werner S., de Vrees L. (2013). Marine Litter within the European Marine Strategy Framework Directive. ICES J. Mar. Sci..

[58] Nava V., Leoni B. (2021). Comparison of Different Procedures for Separating Microplastics from Sediments. Water.

[59] Zobkov M.B., Esiukova E.E. (2017). Evaluation of the Munich Plastic Sediment Separator Efficiency in Extraction of Microplastics from Natural Marine Bottom Sediments. Limnol. Oceanogr. Methods.

